# The Metal-Oxide Nanoparticle–Aqueous Solution Interface Studied by Liquid-Microjet Photoemission

**DOI:** 10.1021/acs.accounts.2c00789

**Published:** 2023-06-13

**Authors:** Hebatallah Ali, Bernd Winter, Robert Seidel

**Affiliations:** † Physics Department, Women Faculty for Art, Science and Education, Ain Shams University, Heliopolis, Cairo 11757, Egypt; ‡ Molecular Physics Department, Fritz-Haber-Institut der Max-Planck-Gesellschaft, Faradayweg 4-6, 14195 Berlin, Germany; § Helmholtz-Zentrum Berlin für Materialien und Energie, Hahn-Meitner-Platz 1, 14109 Berlin, Germany; ∥ Department of Chemistry, Humboldt-Universität zu Berlin, Brook-Taylor-Straße 2, 12489 Berlin, Germany

## Abstract

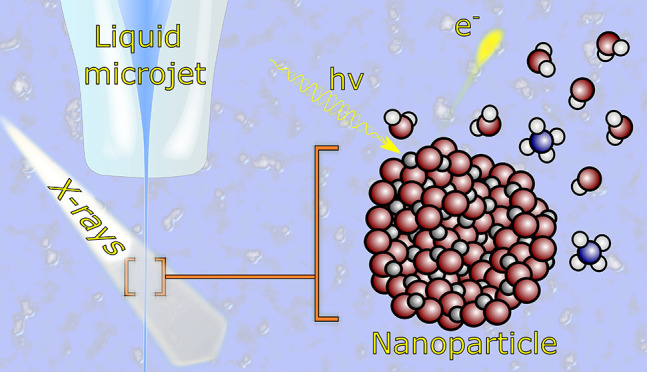

The liquid-microjet technique combined with soft X-ray photoelectron spectroscopy (PES) has become an exceptionally powerful experimental tool to investigate the electronic structure of liquid water and nonaqueous solvents and solutes, including nanoparticle (NP) suspensions, since its first implementation at the BESSY II synchrotron radiation facility 20 years ago. This Account focuses on NPs dispersed in water, offering a unique opportunity to access the solid–electrolyte interface for identifying interfacial species by their characteristic photoelectron spectral fingerprints. Generally, the applicability of PES to a solid–water interface is hampered due to the small mean free path of the photoelectrons in solution. Several approaches have been developed for the electrode–water system and will be reviewed briefly. The situation is different for the NP–water system. Our experiments imply that the transition-metal oxide (TMO) NPs used in our studies reside close enough to the solution–vacuum interface that electrons emitted from the NP–solution interface (and from the NP interior) can be detected.

We were specifically exploring aqueous-phase TMO NPs that have a high potential for (photo)electrocatalytic applications, e.g., for solar fuel generation. The central question we address here is how H_2_O molecules interact with the respective TMO NP surface. Liquid-microjet PES experiments, performed from hematite (α-Fe_2_O_3_, iron(III) oxide) and anatase (TiO_2_, titanium(IV) oxide) NPs dispersed in aqueous solutions, exhibit sufficient sensitity to distinguish between free bulk-solution water molecules and those adsorbed at the NP surface. Moreover, hydroxyl species resulting from dissociative water adsorption can be identified in the photoemission spectra. An important aspect is that in the NP(aq) system the TMO surface is in contact with a true extended bulk electrolyte solution rather than with a few monolayers of water, as is the case in experiments using single-crystal samples. This has a decisive effect on the interfacial processes that can occur since NP–water interactions can be uniquely investigated as a function of pH and provides an environment allowing for unhindered proton migration. Our studies confirm that water is dissociatively adsorbed at the hematite surface and molecularly adsorbed at the TiO_2_ NP surface at low pH. In contrast, at near-basic pH the water interaction is dissociative at the TiO_2_ NP surface.

The liquid-microjet measurements presented here also highlight the multiple aspects of photoemission necessary for a full characterization of TMO nanoparticle surfaces in aqueous environments. For instance, we exploit the ability to increase species-specific electron signals via resonant photoemission, so-called partial electron yield X-ray absorption (PEY-XA) spectra, and from valence photoelectron and resonant Auger-electron spectra. We also address the potential of these resonance processes and the associated ultrafast electronic relaxations for determining charge transfer or electron delocalization times, e.g., from Fe^3+^ located at the hematite nanoparticle interface into the aqueous-solution environment.

## Key References

- AliH.; SeidelR.; PohlM. N.; WinterB., Molecular species forming at the α-Fe_2_O_3_ nanoparticle–aqueous solution interface. Chem. Sci.2018, 9 ( (19), ), 4511–45232989639410.1039/c7sc05156ePMC5961451.^1^*Local electronic-structure interaction, dissociative water adsorption, and electron-delocalization time at the α-Fe_2_O_3_ nanoparticle–aqueous solution interface are revealed by resonant liquid-microjet photoelectron spectroscopy at the oxygen 1s and iron 2p resonances.*
- AliH.; SeidelR.; BergmannA.; WinterB., Electronic structure of aqueous-phase anatase titanium dioxide nanoparticles probed by liquid jet photoelectron spectroscopy. J. Mater. Chem. A2019, 7, 6665–6675.^2^*A pH-dependent mechanism of TiO_2_ nanoparticle–water interaction is proposed based on the observed associated and dissociative water electronic structure on the TiO_2_ nanoparticles surfaces in different chemical environments by resonant liquid-microjet photoelectron spectroscopy.*
- AliH.; GolnakR.; SeidelR.; WinterB.; XiaoJ., In-Situ X-ray Spectroscopy of the Electric Double Layer around TiO_2_ Nanoparticles Dispersed in Aqueous Solution: Implications for H_2_ Generation. ACS Appl. Nano Mater.2020, 3 ( (1), ), 264–273.^3^*Combined liquid-microjet photoemission and photon-emission spectroscopy study provides insight into the composition and the dimension of the electric double layer surrounding TiO_2_ nanoparticles in an aqueous basic solution.*

## Introduction

Knowledge of the electronic-structure interactions at the solid–liquid interface is of large relevance for many fields of technology, especially for the development of novel energy materials,^4^ for our understanding of corrosion and dissolution,^5^ and for photocatalysis,^6^ e.g., (sun)light-induced water-splitting.

**Figure 1:**
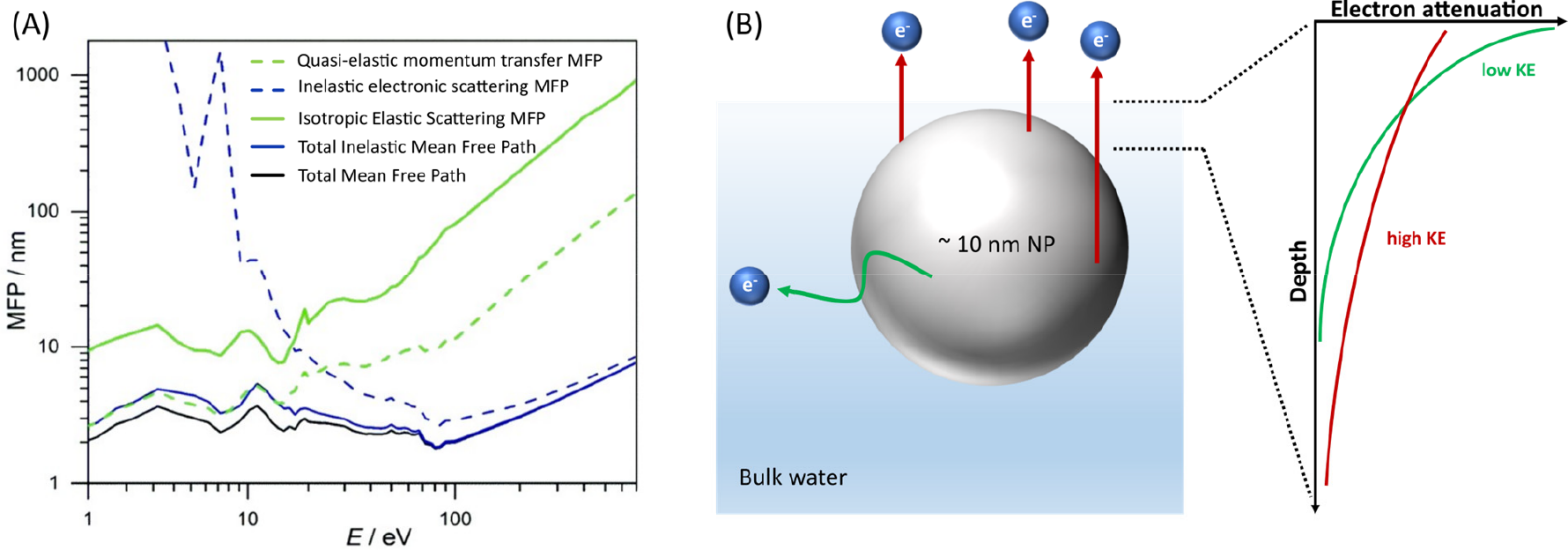
The traveling path of photoelectrons in liquid water depends on their kinetic energy. (A) Mean free paths for electrons in liquid bulk water. (B) Photoelectrons released from the NP into bulk water can escape into vacuum, provided the NP resides near the solution–vacuum interface. The electron attenuation, i.e., the fraction of electrons that escape without any inelastic scattering, is dependent on the kinetic energy of the electrons: the higher the energy, the more electrons from deeper layers can be detected. Adapted with permission from ref (7). Copyright 2020 American Physical Society.

Experimentally, electronic-structure information, and specifically electron binding energies, can be uniquely accessed by photoelectron spectroscopy (PES) from both gas-phase and condensed matter. Yet the application of PES to condensed matter is limited by a rather small information depth of a few nanometers into the sample, determined by the (total) electron mean free path (MFP). In fact, the probing depth is adjustable over some range by variation of the electron kinetic energy (eKE) through the applied photon energy, as depicted in Figure 1A for liquid water, showing on a double-logarithmic scale the latest data on the electron MFP.^7,8^ The important observation is a transition from electronic (ionization, dissociation, excitation) to vibrational inelastic quasi-elastic (meV-loss) scattering channels, the latter contributing to the 10–14 eV eKE range.^9^ There is growing consensus that for eKEs < 100 eV the largest sensitivity for surface probing is obtained, corresponding to approximately eight layers (∼2 nm) of water. For eKEs above 100 eV the probing depth in water increases, e.g., for 700 eV eKE the total electron MFP is ∼6 nm.^7,8^ However, even when using tender X-rays (approximately 2–5 keV^10^) for ionization, the associated total MFP is still too small to detect photoelectrons emitted from a solid sample (e.g., an electrode) fully embedded in bulk (aqueous) solution. Decreasing the solution-layer thickness to match the length scale of the total MFP is currently not feasible. This explains why PES studies from the solid–liquid (aqueous) interface remain challenging, and several alternative approaches have been developed, including, to name a few, (i) solid surfaces prepared under ultrahigh vacuum conditions and subsequently exposed to water molecules via a leak valve,^11^ (ii) freezing water layers on single-crystal surfaces,^12^ (iii) ambient-pressure photoelectron (AP-PE) measurements of solid surfaces covered by few monolayers of liquid water stabilized via relative humidity,^13^ and (iv) liquid cells introduced into the vacuum chamber to record signals through an ultrathin membrane.^14,15^ All these attempts deal with either a membrane barrier or with (too) few molecules or layers of liquid water/aqueous solution on the solid surface, which impair the ion dynamics and reactivity and are far from the realistic solid–bulk solution condition.

A special case of an AP-PE technique is the dip-and-pull technique,^16−18^ where a thin liquid film is prepared by pulling up an electrode from a (degassed) solution beaker within a “vacuum” chamber and stabilized under equilibrated water vapor pressure (∼10 mbar at 10 °C). Films inevitably exhibit a thickness gradient as a function of distance from the solution reservoir.^16−18^ A potential drawback of this technique is that the electrolyte films are still too thin for carrying out practical electrochemical experiments. One concern is that in the case where chemical reactions involve solutes within the thin solution film, solute replenishing would require long diffusion times from the reservoir to the meniscus films. Such a problem would not occur when using a microfluidic cell equipped with a bilayer of graphene. Using such a two-dimensional photoelectron-transparent material, PE spectra from species close to the graphene–water interface have been measured.^19^ However, the graphene membranes are not sufficiently robust, especially when the graphene coverage is not 100%. Depending on the cell design, gas evolution upon X-ray exposure will occur, which can be minimized by using a flow-cell scheme. Furthermore, applying voltages above 1.6 V versus the reversible hydrogen electrode for operando measurements will lead to irreversible graphene oxidation that will lower the membrane stability even further. Note also that with current cell designs, electron spectroscopy studies from a specific desired electrode surface in contact with bulk aqueous solution are not feasible, limited by the fact that photoelectrons must traverse the electrode material and the cell membrane.

Here we review and discuss a different approach, mimicking the solid–liquid interface and probing its electronic structure, namely, liquid-microjet PES (LJ-PES)^20,21^ from NPs dispersed in aqueous solution.^1−3^ In the NP solutions considered in this Account, hematite (α-Fe_2_O_3_, iron(III) oxide) and anatase (TiO_2_, titanium(IV) oxide) NPs in water, some fraction of the NPs reside close enough to the solution–vacuum interface that electrons emitted from the NP–solution interface and even from the NP interior reach and cross the solution–vacuum interface and can be detected. This is illustrated in Figure 1B, showing that the experimental probing depth is large enough to detect electrons emitted from the solvent, the NP–solution interface, and even the NP (near-surface) interior. For our studies we used soft-X-rays to ionize and electronically excite the NP aqueous solutions. An advantage of fast-flowing liquid microjets is their insensitivity to beam damage because of the fast material replenishing and the short exposure time to the environment, also minimizing contaminations of the liquid.^20,21^ In addition to detecting direct photoelectrons, also resonant photoemission was applied, which leads to unique resonant Auger electron fingerprints that increase valence-orbital-specific electron signals, as we will explain. We briefly note here the large potential of FTIR measurements for identifying hydroxylated and hydrogenated surface species.^22−24^ The present Account yet focuses on the associated electron energetics accessed from photoemission.

Although the photoemission spectra from NP solutions appear to capture well the occurring molecular species at this interface as well as helping to advance our understanding of the conditions and mechanism for water dissociation, the approach lacks the ability to directly measure the transition-metal oxide (TMO) surface structure in solution. This is furthermore impeded by the fact that some fraction of the NP surface must be covered by suitable molecular stabilizers to hinder NP aggregation. In our studies, we dispersed the NPs in inorganic aqueous solutions containing Cl^–^, NH_4_^+^, and NO_3_^–^ ions, respectively, which adsorb at the NP surface. Only when the surface of the nanoparticles is charged can they repel each other and be stabilized in solution. The challenge thus is to charge the surface of the NPs while providing enough free surface sites for the liquid water molecules to interact. To solve this, we performed several explorative studies, varying the NP size and their concentration relative to the concentration of the stabilizer ions. In that way we also vary the zeta potential and the associated solution pH to capture changes in the surface chemistry as a function of the latter.

## Experimental Section

### Hematite and Anatase

Experiments reported here have been performed with both hematite (α-Fe_2_O_3_) and anatase (TiO_2_) NPs due to their potential use in photo(electro)catalytic water splitting^25−27^ as well as their role in many technological, environmental, and biological applications.^28^ Both materials are inexpensive and abundant. Hematite is the thermodynamically most stable iron oxide and has a band gap of 2.2–2.7 eV^29^ (i.e., it absorbs visible light). During the catalytic processes, the TMO surfaces directly interact with liquid water or electrolytes. This has led to several theoretical and experimental studies to determine the surface structure and termination and to unveil the nature of water interaction with these surfaces in the absence of an applied potential and without photoactivation.^30^ There is an overall consensus in the literature that water interacts dissociatively with hematite, regardless of its surface termination. However, it should be noted that all the experimental investigations so far have studied the surface interaction with few molecules of water vapor only, with the exception of one study from Yamamoto et al. using AP-PE spectroscopy.^13^ These latter authors concluded that hydroxyl groups accommodate within the first monolayer on the iron oxide surface, and when the relative humidity is increased, molecular water starts to form the second monolayer on the surface. Note that the few-water-monolayer system stabilized by relative humidity is unsuited for variation of pH.

Anatase is the most active and abundant form of TiO_2_. It absorbs UV light due to its wide band gap of 3.2 eV and has been intensively studied experimentally^31^ and theoretically.^32^ (Doped) TiO_2_ is used as photoanode material in photoelectrochemical cells for solar hydrogen generation, but its solar-to-hydrogen conversion efficiency is much lower than the desirable threshold for industrial and commercial applications due to the unwanted back-reaction (i.e., catalytic water formation).^33^ There are three proposed mechanisms for the TiO_2_–water interaction reported in the literature. In mechanism (1), water interacts dissociatively with TiO_2_ defect surface sites. These defects correspond to a missing oxygen atom in the TiO_2_ surface crystal structure associated with a reduction from Ti^4+^ to Ti^3+^.^34^ Mechanism (2), for a defect-free surface, involves the molecular water adsorption at the surface, where the oxygen atom of water binds to Ti^4+^ and the water hydrogen atoms bind to neighboring lattice oxygen atoms.^35^ Mechanism (3) assumes a mixed adsorption behavior identifying ∼0.47 monolayer of OH versus ∼0.8 monolayer of H_2_O in the first water monolayer on a defect-free anatase TiO_2_(101) surface.^36^ Our own findings from LJ-PES from TiO_2_ NP aqueous solutions to be presented below are consistent with mechanism (3).

### Stabilization of Hematite and Anatase NPs in Aqueous Solution

**Figure 2:**
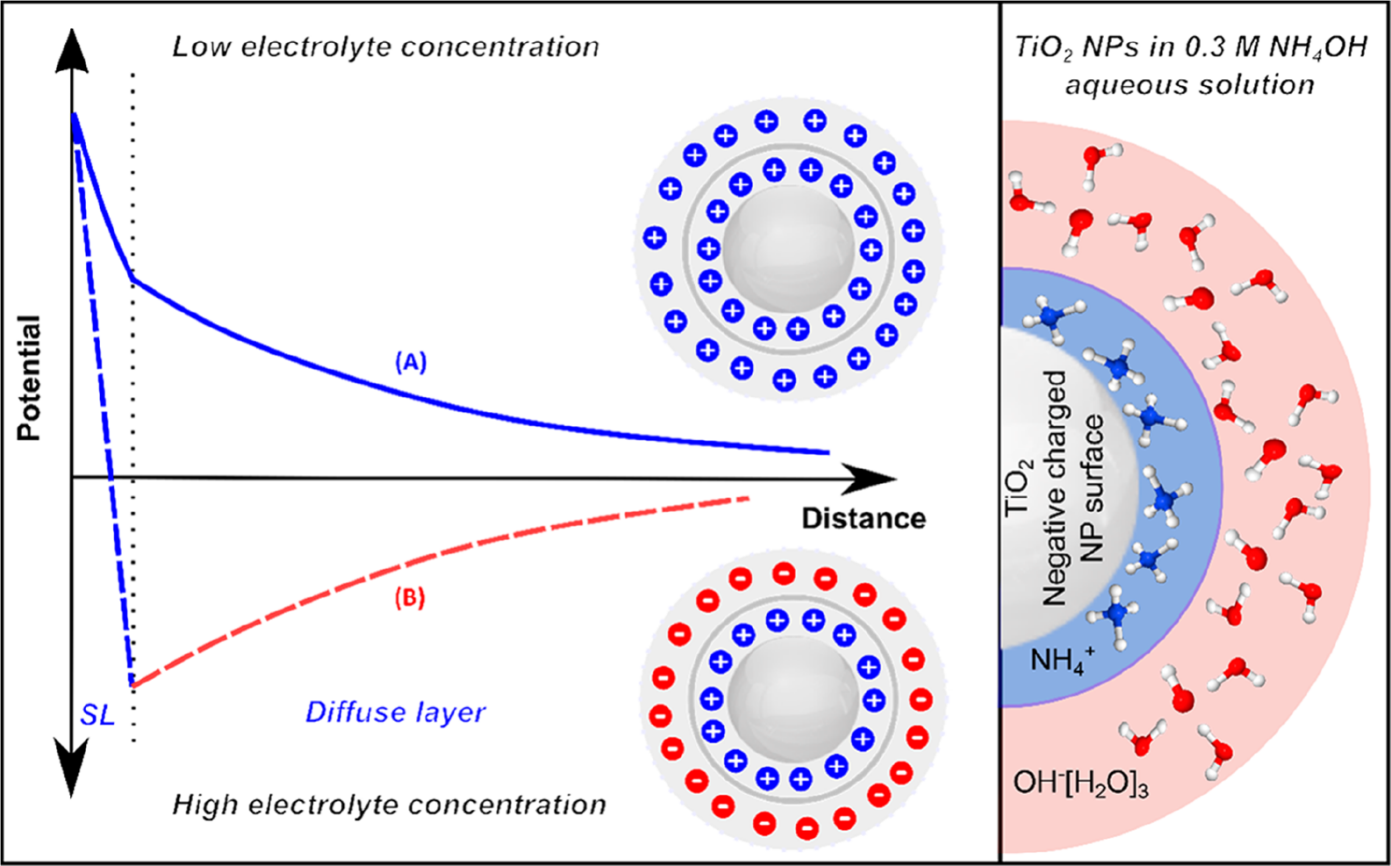
The potential curve of the electric double layer (EDL) around a nanoparticle depends on the electrolyte concentration. For high electrolyte concentrations, exemplified for TiO_2_ NPs in 0.3 M NH_4_OH aqueous solutions, NH_4_^+^ counterions are adsorbed at the NP surface, forming the Stern layer (SL). In contrast to low electrolyte concentration, the diffuse layer has reversed parity relative to the SL. Reproduced from ref (3). Copyright 2019 American Chemical Society.

Dispersing NPs in water inevitably results in their aggregation, and this can be avoided by charging the NP surface by adding suitable stabilizing ions, as depicted schematically for the case of TiO_2_ NPs in 0.3 M NH_4_OH aqueous solution in Figure 2. The NH_4_^+^ co-ions are chemically adsorbed on the surface of the nanoparticles, forming a Stern layer.^37^ This layer is followed by the diffuse layer, in which the concentration of mobile ions follows the Boltzmann distribution. Together, the Stern layer and the diffuse layer form the electric double layer (EDL); the full length of the EDL is called the Debye length. Depending on the type and concentration of the electrolyte, the stability and the parity of the EDL vary according to the theory of Derjaguin, Landau, Verwey, and Overbeek (DLVO).^38−40^ The DLVO theory describes the net electrostatic force between the NPs as a function of their mutual distance. It is the sum of the Coulombic repulsion by adsorbed ions and the van der Waals attraction between the free sites on the NPs surfaces. At low electrolyte concentration, the parities of the Stern layer and the diffuse layer are the same; see curve (A) in Figure 2. At high electrolyte concentration, which is the case in the present study, the parity of the diffuse layer is reversed relative to that of the Stern layer; see curve (B) in Figure 2. At charge balance (zero net charge), i.e., at the isoelectric point (IEP), the repulsive electrostatic forces are reduced, and the attraction forces predominate, causing NP aggregation and precipitation. In addition, the IEP is the pH value at which the zeta potential value is zero, implying no electric charge on the surface of a particle. It is the electric potential at the boundary between the Stern layer and the diffuse layer and relates to the mobility of the NP in solution; the square of the zeta potential is proportional to the force of electrostatic repulsion between two charged particles. The zeta potential thus senses the specific surface chemistry of a given NP solution, affected by changes in pH and salt (stabilizer) concentration. At solution pH that is above the IEP, the surface of the NP is predominantly negatively charged; this is the case for the stabilization of an anatase NP dispersion by NH_4_^+^, with a pH value larger than the IEP (close to 6). We measured TiO_2_ NPs in several solutions using different stabilizing ions and concentrations, with different particle diameters ranging from 3 to 20 nm, and at different pH values; see Table 1 for an overview of all investigated NP solutions. That table also has an entry of the available and experimentally variable free surface sites for a given NP dispersion system, correlating with the stability in solution through electrostatics. These values can be determined from an estimate of the surface area of all contained NPs and the stabilizer concentration.

**Table 1:** Summary of the NP Sizes, NP Concentrations, Molecular Stabilizers Used, Their Respective Concentrations, Resulting pH of the Solutions, and the Ratio of Free Surface Sites to Stabilizer Molecules for Our Liquid-Microjet Photoelectron Measurements

NP composition	NP size (nm)	NP conc. (wt %)	stabilizer ion	stabilizer conc. (M)	pH	[*x*:*y*]^co-ion^^a^
anatase TiO_2_	10	20	Cl^–^	1.00	0.70	[1:2]^Cl^–^^
				0.50	1.20	[1:1]^Cl^–^^
	6		NO_3_^–^	0.50	1.20	[1:1]^NO_3_^–^^
				0.25	0.90	[2:1]^NO_3_^–^^
	3			0.60	0.70	[4:1]^NO_3_^–^^
	20		NH_4_^+^	0.30	7.80	[2:1]^NH_4_^+^^
hematite α-Fe_2_O_3_	6	5	NO_3_^–^	0.10	1.55	[1:1]^NO_3_^–^^
		10		0.10	1.90	[2:1]^NO_3_^–^^
				0.05	2.00	[4:1]^NO_3_^–^^

a *x*:*y* denotes the ratio of surface sites to stabilizer ions, and the superscript indicates the stabilizer ion used.

In the case of hematite, the NP diameter was 6 nm, and the concentration of the NPs as well as the stabilizing NO_3_^–^ co-ions, from HNO_3_ added to the aqueous solution, was varied. We dispersed 5 wt % 6 nm hematite NPs in 0.1 M aqueous HNO_3_ solution (pH = 1.55) and 10 wt % NPs in 0.1 M aqueous HNO_3_ solution (pH = 1.9) and in 0.05 M aqueous HNO_3_ solution (pH = 2). It is reminded that pH is not an independent variable in all our experiments but rather results from the stabilizer and its concentration used. For our experiments it is crucial that the NPs can be stabilized at a surface coverage well below monolayer coverage, which we determine from the NP and stabilizer concentrations and from the NP bulk and surface density. The first solution of Table 1 contains NPs that have a surface completely covered with NO_3_^–^, denoted as [1:1], where no free surface sites are available for water interaction, while the second and the third solutions exhibit NP surfaces that contain 50%, [2:1], and 25%, [4:1], NO_3_^–^ coverage, respectively. The “[4:1] solution” has the highest ratio of free hematite surface sites to stabilizer that we could realize to run a stable vacuum laminar liquid microjet. The anatase NPs studied had diameters of 3, 6, 10, and 20 nm. We prepared aqueous solutions containing 20 wt % anatase NPs stabilized by three different anions, Cl^–^, NO_3_^–^, and NH_4_^+^. Two Cl^–^-stabilized samples are acidic solutions without free surface sites, and the TiO_2_ NPs are fully covered [1:1]^Cl^–^^ and doubly covered [1:2]^Cl^–^^. They are used as a reference for the signal from TiO_2_ aqueous NPs without water interaction or interfacial oxygen species. All NO_3_^–^-stabilized samples are acidic solutions with free surface sites where the TiO_2_ surface sites to stabilizer ions ratio changes from [1:1]^NO_3_^–^^ to [2:1]^NO_3_^–^^ and [4:1]^NO_3_^–^^. In the [4:1]^NO_3_^–^^ solution, 75% of the TiO_2_ surface sites are interacting with liquid water. In addition, we explored stabilization by 0.3 M NH_4_OH, resulting in a basic solution (pH = 7.8) with a free-surface-site ratio of [2:1]^NH_4_^+^^, i.e., approximately half of the TiO_2_ surface sites are available for a reaction with water.

## Liquid-Microjet PES Measurements

The following paragraphs review several important findings for anatase and hematite NP aqueous solutions. Our focus is on core-level PE spectra and the potential of resonant valence PES for detection of low signal intensity from the NP–solution interface that would otherwise stay undetected. We also discuss the sensitivity of partial electron yield (PEY) measurements to distinguish between bulk and interfacial electronic structure as well as to provide insight into the electron delocalization, exemplified for hematite, from core-excited iron into its aqueous-phase environment. All LJ-PES studies were conducted at the undulator beamline U49/2-PGM-1 at the synchrotron radiation facility BESSY II, Berlin, using the SOL^3^PES setup.^41^

### Anatase NP–Water Interface

**Figure 3:**
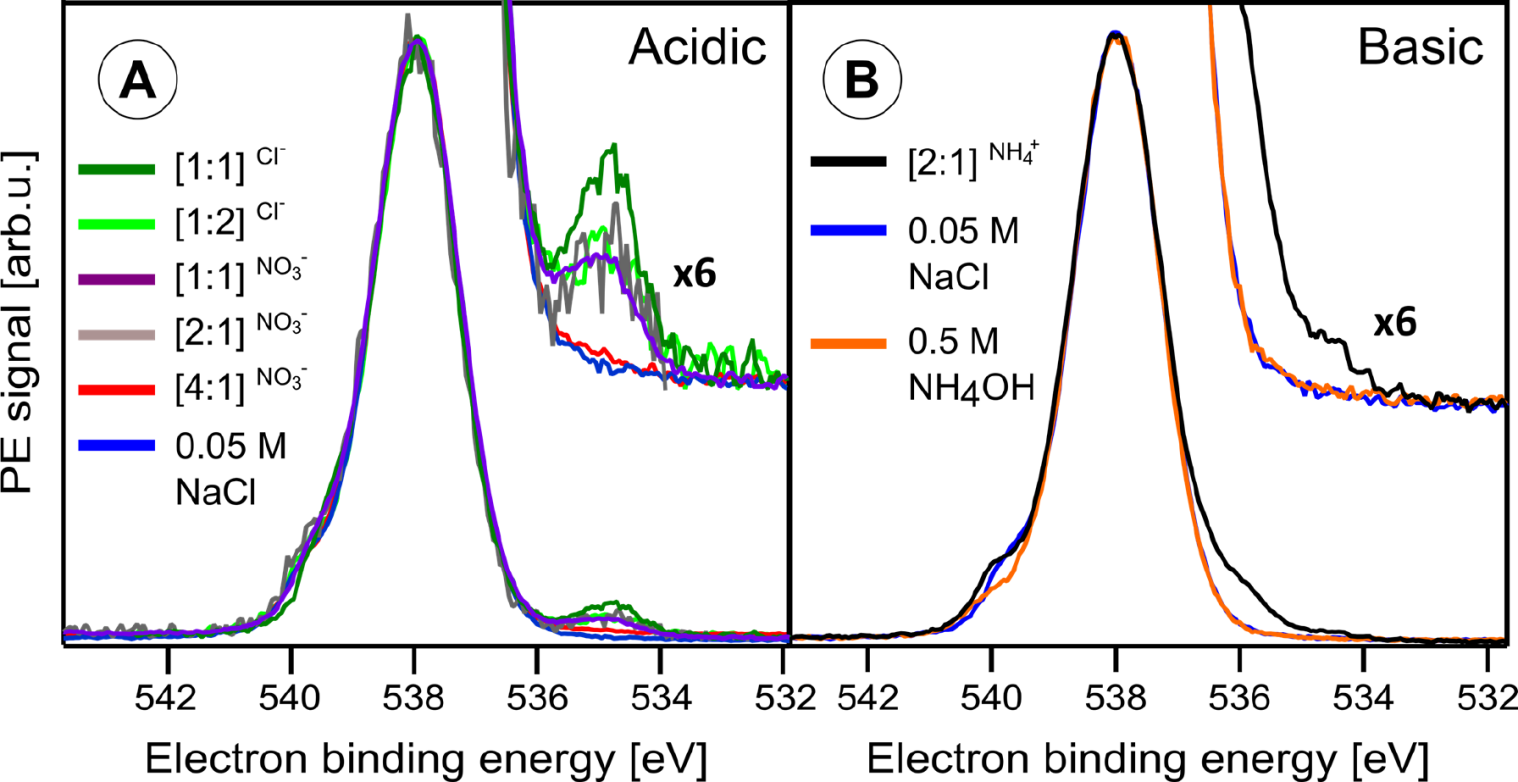
Oxygen 1s photoelectron spectra of different anatase TiO_2_ NP aqueous solutions in (A) acidic and (B) basic pH, measured at 1200 eV photon energy. [*x*:*y*]^ion^ indicates by the superscript the stabilizer ion used, and the brackets state the free TiO_2_ surface sites to stabilizer ratio. Also shown are the O 1s spectra from 0.05 M NaCl and 0.5 M NH_4_OH aqueous solutions. From ref (2). CC BY 3.0.

**Figure 4:**
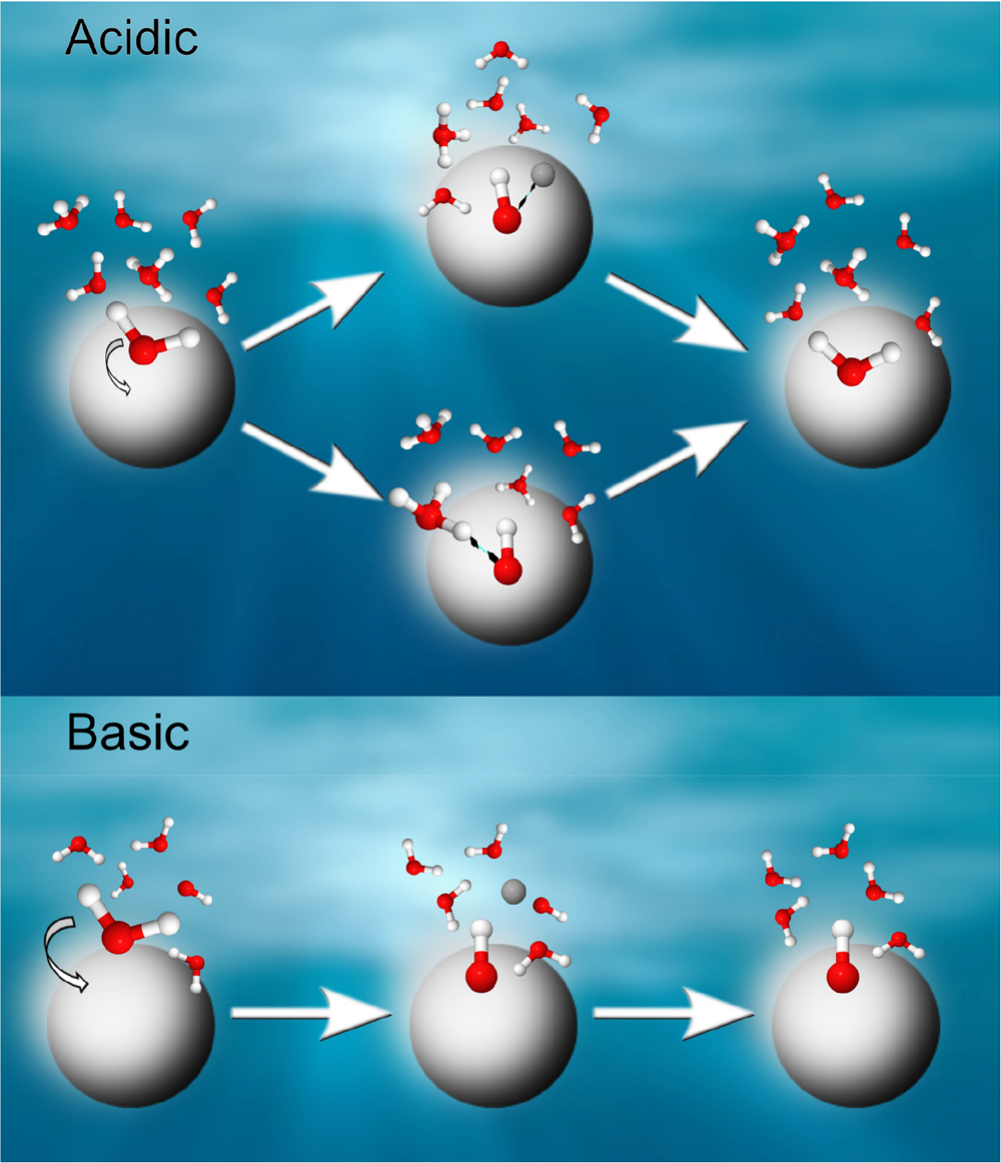
Proposed TiO_2_ NP–water interaction in acidic (top) and basic (bottom) aqueous solutions. Water, hydroxide, and hydronium oxygens are shown in red, bonded hydrogen atoms are shown in white, and a single free hydrogen (proton) in solution is shown in gray. The hydroxyl stability on the NP surface depends on its probability of forming a water molecule by capturing a free H^+^ or via proton transfer from a surrounding hydronium. This probability is largest in the acidic environment, either by recombination of the dissociated H^+^ and OH^–^ pairs or by proton transfer from the surrounding hydronium. Such recombination and proton transfer processes do not occur in a basic chemical environment. From ref (2). CC BY 3.0.

Exemplified for 20 wt % anatase TiO_2_ NP aqueous solutions, Figure 3 presents the regular oxygen 1s PE spectra obtained for different surface free areas (adjusted by stabilizer concentration, i.e., surface site-to-stabilizer ratio), with Figure 3A referring to acidic pH and Figure 3B to near-neutral solution pH (compare Table 1). Spectra from neat water (0.05 M NaCl added to increase the conductivity^20^) are shown as well for reference. Measurements were performed at 1200 eV photon energy, i.e., O 1s photoelectrons with kinetic energies of about 650 eV are generated, which corresponds to probing approximately 5.5 nm into neat liquid water (compare Figure 1A) and sufficiently deep into the NP solution to even detect TiO_2_ PES signal (compare Figure 1B). In fact, the spectra identify the main oxygen-containing species from the NP–solution interface. The peak at 538.1 eV corresponds to the O 1s binding energy of liquid water, the asymmetric shoulder at 540 eV binding energy represents the oxygen 1s signal of water vapor, and the peaks at 536.0 and 534.7 eV binding energy are assigned to OH^–^ and TiO_2_ lattice oxide, respectively. We note that NO_3_^–^(aq) is undetectable due to its overlap with the liquid water peak at 538.1 eV binding energy. Due to missing OH^–^ signal under the conditions of Figure 3A, we conclude that water is molecularly adsorbed, i.e., not dissociated at the TiO_2_ surface at acidic pH. On the other hand, at pH 7.8 (Figure 3B), we observe a large OH^–^ photoelectron signal contribution at 536.0 eV binding energy for the [2:1]^NH_4_^+^^ TiO_2_ NPs aqueous solution. It is important to note that no OH^–^ signal is observed from a 0.5 M NH_4_OH aqueous solution of pH 11.7 (containing no NPs) because ∼10^–2^ M free OH^–^ is below our detection limit. It is therefore surprising to observe a very strong OH^–^ signal in the [2:1]^NH_4_^+^^ NPs aqueous sample at even lower pH, with only 10^–7^ M free OH^–^ from self-ionization of water. As we propose below, this large OH^–^ signal arises from the dissociative water interaction with the TiO_2_ NPs free surface sites, with OH^–^ staying trapped around the NPs. At the defect-free TiO_2_ surface, water dissociates into H^+^ and OH^–^,^36^ the latter detaching from the surface and chemically interacting with the surrounding species. Whether OH^–^ is being stabilized depends on the availability of nearby H^+^ species, as previously detailed using DFT calculations.^42^ In an acidic environment, on the other hand, the recombination of OH^–^ with a short-lived free proton H^+^, which is locally confined due to surrounding charges, is likely to happen. Alternatively, a proton transfer from hydronium would also be possible. In both cases, the OH^–^ species will not last long enough to be detected with our spectroscopic techniques. On the other hand, at above-neutral pH, the OH^–^ and H^+^ would diffuse sufficiently away from each other, increasing the probability of hydroxyl species to survive. This is illustrated in Figure 4 for the acidic (top) and basic (bottom) environments. The left parts show an initially intact water molecule attached to the TiO_2_ NP in both environments. The respective center images show dissociated water along with the mentioned recombination processes to form water in the acidic case, and the final products are shown at the right.

**Figure 5:**
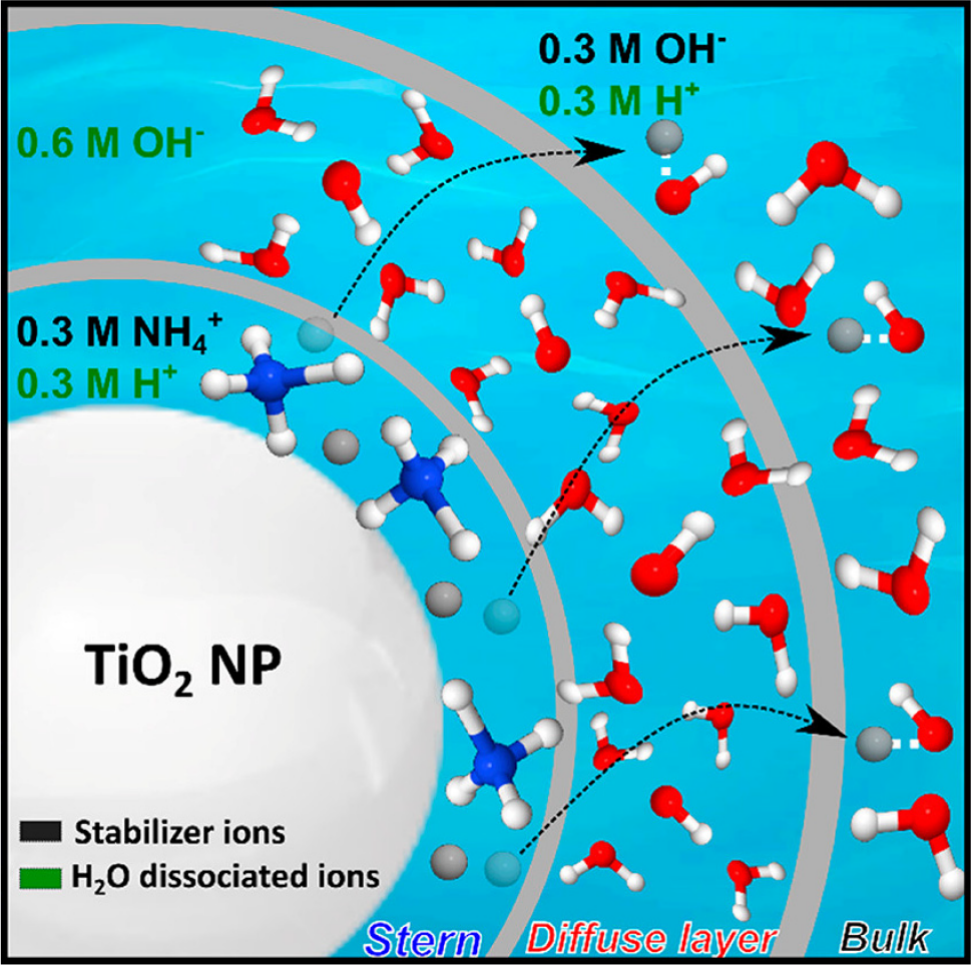
Schematic representation of the EDL and its formation around TiO_2_ NPs in 0.3 M NH_4_OH aqueous solution. H^+^ (gray spheres) from the water dissociation partially escape into the bulk solution, preventing unwanted H^+^–OH recombination. From the quantitative analysis of complementary partial fluorescence yield XAS measurements (see ref (3), not further detailed here), enabling probing of the bulk aqueous NP solution, we determined that 0.6 M H_2_O is dissociated, which creates 0.6 M OH^–^ ion in the diffuse layer. Given that at 0.3 M NH_4_^+^ concentration approximately half the TiO_2_ NP surface sites are covered, 0.6 M OH^–^ would be enough to exceed one monolayer coverage, forming the diffuse layer. Of the corresponding 0.6 M H^+^, approximately half of the H^+^ are bound to the TiO_2_ surface, and the other fraction quickly diffuse into the bulk solution, neutralizing the solution. Adapted from ref (3). Copyright 2019 American Chemical Society.

However, these considerations are insufficient to explain the apparent discrepancy of the large OH^–^ signal at the slightly basic pH of 7.8 (Figure 3), which requires insight into the structure and the dimension of the EDL surrounding the NPs sample. Referring to Figure 2, at high electrolyte concentration, in the case of the 0.3 M NH_4_OH stabilizer concentration, the EDL consists of a Stern layer where the NH_4_^+^ counterions are accumulated at the surface and a diffuse mobile layer where the co-ions are diffusing away from the Stern layer. Based on a combination of oxygen K-edge partial fluorescence yield and nitrogen K-edge partial electron yield X-ray absorption (PEY-XA) as well as nitrogen 1s photoelectron measurements, as exemplarily shown for the nitrogen spectra in Figure S1 (from ref (3)) for the [2:1]^NH_4_^+^^ TiO_2_ solution (corresponding to 50% freely available TiO_2_ surface sites for water molecules), we have concluded that most of the OH^–^ molecules in the NP solution must be trapped around the TiO_2_ NPs by the associated positive Stern layer, forming the diffuse layer (estimated 0.8 nm thick) of the EDL. Importantly, these confined OH^–^ species make no contribution to the pH measurement, thus explaining the unexpectedly low pH value; an estimate of the molar OH^–^ concentration is provided in the caption of Figure 5. Specifically, because of the limited number of anchoring oxygen sites for water on the TiO_2_ surface, the excess of OH^–^ can be rationalized when assuming that some fraction of H^+^ ions produced at the interface migrate through the diffuse layer, recombining with the original free OH^–^ from the 0.3 M ammonia solution to form water. The other fraction of H^+^ will however inevitably recombine with OH^–^ within the diffuse layer, and the resulting loss of OH^–^ molecules can be replenished because the H^+^ release from the TiO_2_ surface vacates adsorption sites for further water dissociation reaction, which in turn generates additional OH^–^ in the diffuse layer. This proposed mechanism is illustrated in Figure 5 along with details on the relevant partial concentrations. Even if we do not understand the detailed mechanism of such efficient proton transfer through the diffuse layer, OH^–^ in the bulk solution as well as in the diffuse layer must play an important role in initiating the release of H^+^ from the TiO_2_ surface. This dynamical cycle of continuous freeing of surface sites for water adsorption and the subsequent release of H^+^ followed by recombination will reach an equilibrium once the bulk solution is nearly neutralized.

### Hematite NP–Water Interface

**Figure 6:**
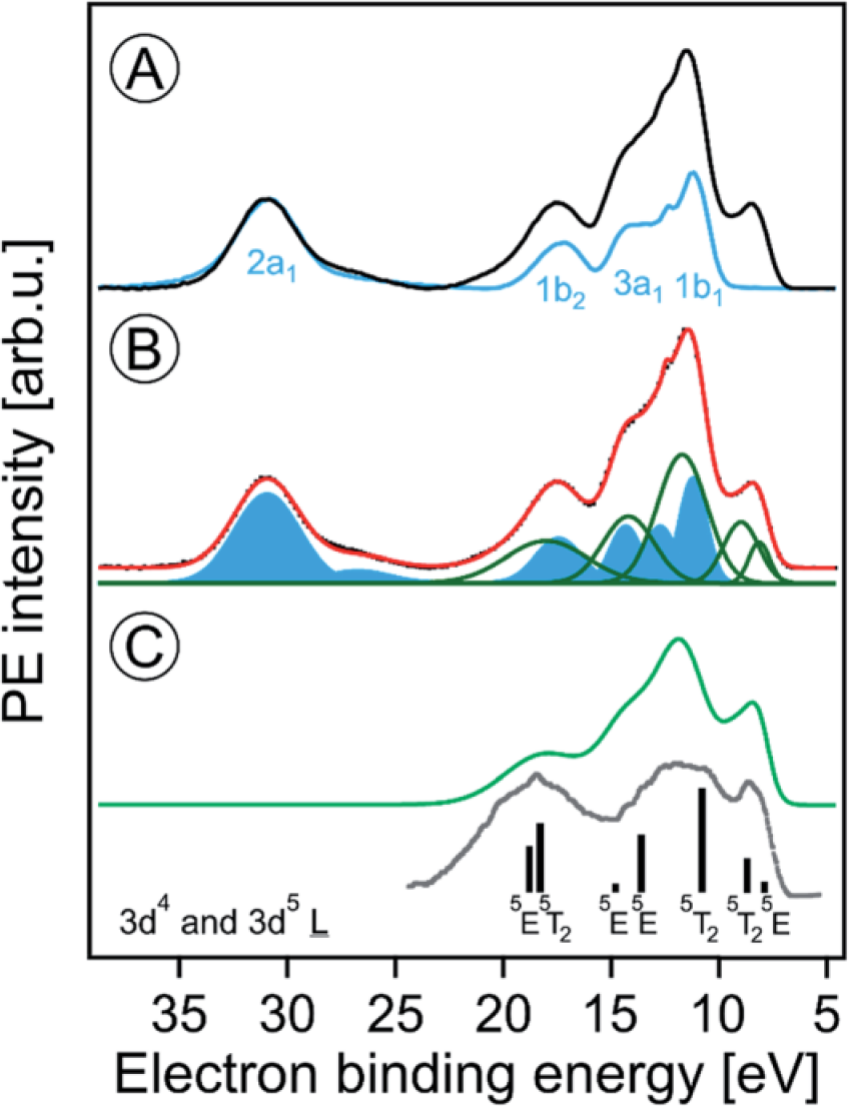
(A) Valence PE spectra from a 5 wt % hematite NP aqueous solution measured at the Fe 2p_3/2_ → VB resonant excitation energy of 710.5 eV (black) and at the off-resonant energy of 704.5 eV. A background has been subtracted in both spectra. Water orbital contributions are labeled in blue. (B) Decomposition of the black resonant PE spectrum from (A) into the off-resonant water contributions (blue Gaussians) and the resonant PE contributions from iron (green Gaussians). (C) The green curve represents solute-only spectral contributions and is the sum of the green Gaussians in (B). Spectral differences to the measured PE spectrum from solid hematite in ultrahigh vacuum (from ref (45)) reveal strong iron 3d–oxygen 2p hybridization that causes a ligand-to-iron charge transfer between water and iron at the NP surface. From ref (1). CC BY 3.0.

**Figure 7:**
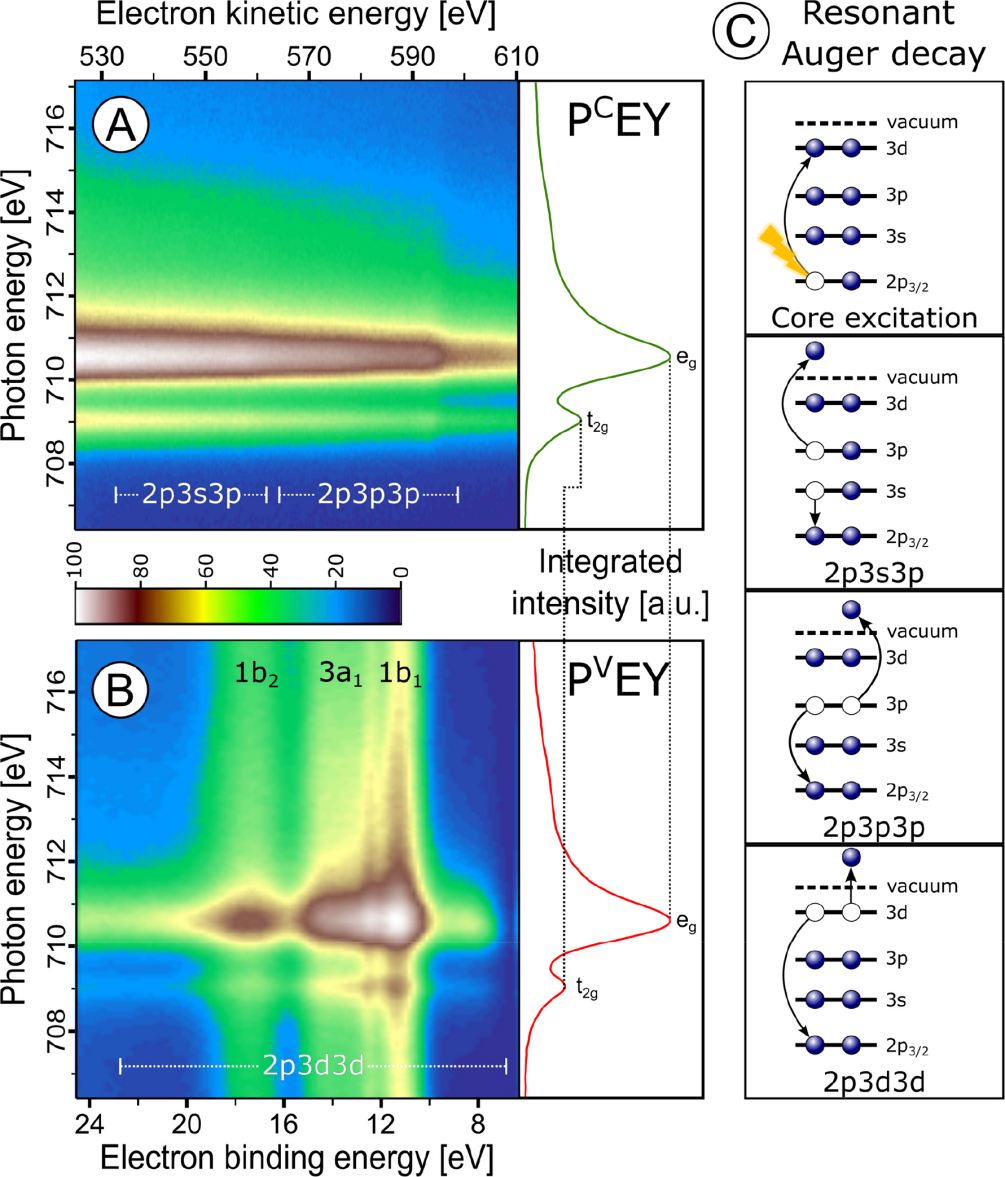
(A, B) Contour plots of the Auger signal intensities from a 10 wt % hematite NPs in 0.1 M HNO_3_ aqueous solution, detected near the Fe 2p_3/2_ to valence excitation energy: (A) presents the measured core-to-core relaxation, while (B) shows the valence-to-core relaxation, as detailed in (C). The green and red curves in (A) and (B) are the resulting Fe 2p_3/2_ partial electron yield (PEY) X-ray absorption spectra.

We first consider the valence PE spectra from a 5 wt % hematite NP aqueous solution, measured off-resonantly at 704.5 eV photon energy (blue spectrum in Figure 6A) and at 710.5 eV (black spectrum), which is the resonance energy to excite an Fe 2p_3/2_ electron into the e_g_ valence level. In both spectra, a Shirley background has been subtracted, and the relative intensities of the two spectra are normalized to the 2a_1_ peak of water, which is the inner-valence peak that remains unaffected by the resonant excitation. The off-resonant blue spectrum in Figure 6A is essentially the spectrum of neat water, not exhibiting solute signal. This absence of the low-energy metal-related 3d emission (the e_g_ and t_2g_ bands near 8.5 eV binding energy) in the off-resonant spectrum directly illustrates the strength of resonant PES (RPES). The large signal enhancement in RPES results from the coherent superposition of the outgoing electron waves for two different channels, direct photoionization and resonant Auger decay (see Figure 7C for more details).^43,44^

Figure 6B presents a fitting analysis of the resonant PE spectrum from Figure 6A. Using the known peak positions and widths of the water valence features^46^ (blue-shaded contribution) we can determine the additional spectral contributions from ionization of the NP (green curves).^1^ This latter signal well resembles the previously reported valence PE spectrum of crystalline hematite measured in ultrahigh vacuum, reproduced in Figure 6C (gray curve). The extra PE signal near 14.5 eV binding energy in the aqueous-phase spectrum arises from the Fe 3d–O 2p hybridization with water and hydroxide which causes a strong ligand-to-iron charge transfer^43^ at the hematite NP surface. We refer to Figure S2 and to ref (1), where we also discuss spectral fingerprints for water dissociation on hematite NPs, as concluded from valence band spectra measured at the oxygen 1s core-level excitation.

Additional electronic-structure information can be obtained from PEY-XA spectra, which are the integrated PE intensities in a specific energy region that contains resonant Auger electron peaks. In Figure 7 we present two contour plots of a series of resonant PE spectra for 10 wt % hematite NPs in 0.1 M HNO_3_ aqueous solution for varying photon energies between 706 and 716 eV near the Fe L_3_-edge. Signal integration of the individual PE spectra results in the PEY-XA spectra shown on the right-hand side of the contour plots. P^C^EY-XA and P^V^EY-XA refer to integration over the core-level (iron 2p3p3p and 2p3s3p resonant Auger electron decay) and valence (iron 2p3d3d Auger electron decay region) spectral regions, respectively.^1^ These different transitions are depicted in Figure 7C. The crucial aspect is that while the 3d orbitals carry information on the mixing with the ligand-centered orbitals, the 2p3p3p and 2p3s3p integration (P^C^EY) does not contain any ligand-interaction contributions and should therefore be a closer representative of a true absorption spectrum measured in transmission.^47^ Both PEY-XA spectra in Figure 7 exhibit two peaks at ∼709 and 710.5 eV photon energy representing the Fe^3+^ transitions 2p_3/2_ → 3d t_2g_ and 2p_3/2_→ 3d e_g_, respectively. When normalizing the PEY-XA spectra to the 710.5 eV peak height, we observe a lower signal intensity in the prepeak for the P^V^EY XA spectrum. This is due to the quenching of the 2p3d3d Auger decay channel, where the excited 2p_3/2_ electron in the 3d valence band delocalizes during the ∼1.8 fs iron 2p core-hole lifetime^48^ and has therefore a lower probability to refill the 2p core hole. There is considerable mixing with the water lone-pair orbitals as well as with the NP lattice oxygen 2p bands. This mixing varies in strength for the e_g_ and the t_2g_ resonances. We also refer to Figure S3 (from ref (1)), which compares the spectra in Figure 7 with PEY-XA spectra from Fe^3+^ monomers in 1 M FeCl_3_ aqueous solution and with a total electron yield (TEY) XA spectrum from solid hematite to extract further information from the ligand-field splitting and the relative charge-transfer probability between iron and oxygen ligands.

**Figure 8:**
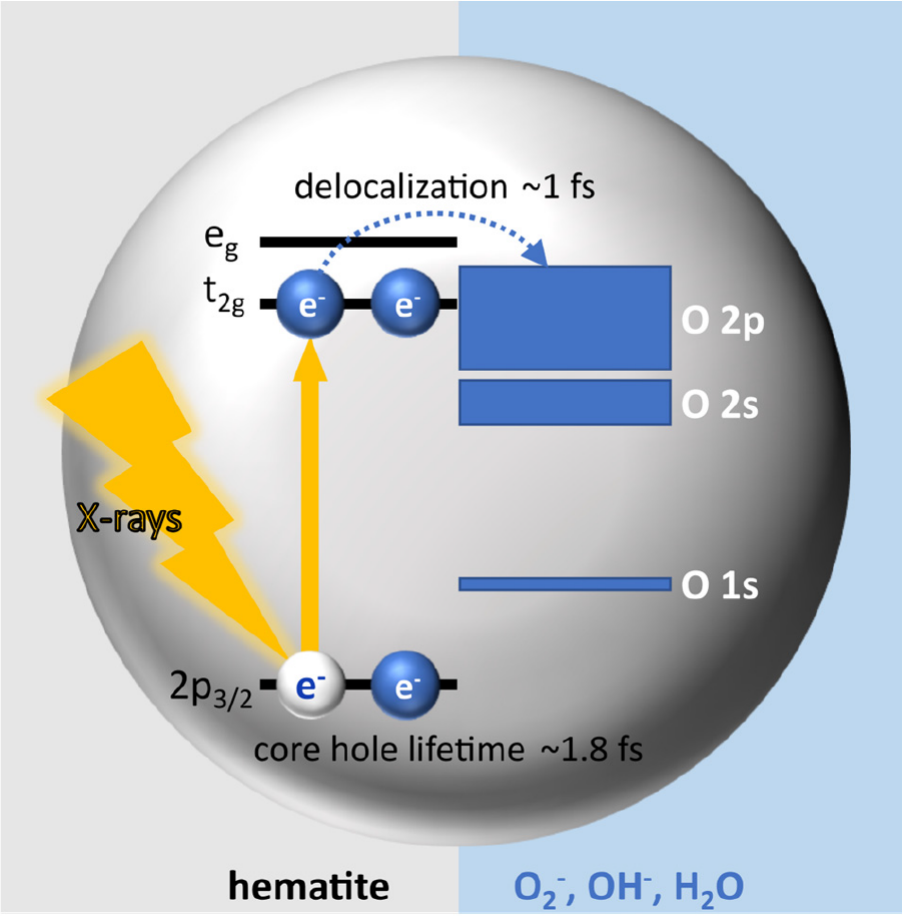
Identifying the ratio of local and nonlocal (Auger decay) contributions to the Fe 2p PEY-XA spectra from hematite NP(aq) enables the determination of the electron delocalization time of core-excited Fe^3+^ into the surrounding aqueous solution. The process requires suitable orbital overlap, as depicted.

We also use the PEY-XA spectra to quantify the charge transfer, or electron delocalization rate, between hematite NPs and solution, specifically from the t_2g_ orbital into the surrounding oxygen orbitals (see Figure 8). This charge-transfer time (τ_CT_) is controlled by the Fe 2p_3/2_ core-hole lifetime, τ_core_, and by the exponential electron delocalization.^49^ It can be expressed by τ_CT_ = τ_core_(*f*_Auger_^–1^ – 1), where *f*_Auger_ is the ratio between normal Auger electron signals and signals from nonlocal decay processes. We extracted this ratio from the t_2g_ XA peak areas from the P^V^EY- and P^C^EY-XA spectra in Figure 7. With *f*_Auger_ = 0.6, and τ_core_ = 1.8 fs from ref (48) (another work has reported τ_core_ = 1.6 fs^50^); we then calculate τ_CT_ ∼ 1 fs. To our knowledge, this quantity has not been experimentally revealed by other techniques, as it would require subfemtosecond laser pulses. Similar measurements could not have been done for TiO_2_ NPs because Ti^4+^ has no valence 3d electrons, and hence, resonant 2p3d3d Auger decay (i.e., P^V^EY-XAS) is not possible.

## Concluding Remarks and Outlook

We have demonstrated the unique potential of liquid-jet photoemission spectroscopy to characterize the electronic structure of Fe_2_O_3_ and TiO_2_ nanoparticle–aqueous solution interfaces as a function of pH. Specifically, the technique is capable of quantifying water dissociation at the NP(aq) surface to identify OH^–^ recombination with H^+^ and characterize the associated electric double layer. Exploiting the various aspects of photoemission, including the detection of direct photoelectrons as well as Auger electrons and associated electron yield absorption spectra, we also identified NP electronic structure changes arising from the interaction with the aqueous solution. Our experiments furthermore allow quantification of an ultrafast electron delocalization from the NP into the aqueous environment. In the longer perspective, it is desirable to apply this set of spectroscopic tools to the electrode–electrolyte interface under operando conditions, as typically realized with a (photo)electrochemical cell. However, further development of suitable sample designs is needed for compatibility with the short electron probing depth in water and aqueous solutions.

## Supporting Information Available

N 1s PEY-XAS and N 1s PES data for TiO_2_ [NH_4_^+^] NPs, resonant photoelectron spectra of α-Fe_2_O_3_ NPs aqueous solutions, and PEY-XAS of hematite NPs (PDF)
