# Supplementary material for: The Metal-Oxide Nanoparticle–Aqueous Solution Interface Studied by Liquid-Microjet Photoemission
Source: Acc Chem Res. 2023 Jun 13;56(13):1687–97. doi: 10.1021/acs.accounts.2c00789 (PMC10324314; doi:10.1021/acs.accounts.2c00789)
Supplement: Supplementary file 1 — ar2c00789_si_001.pdf [file ar2c00789_si_001.pdf]

Supporting Information

The Metal-Oxide Nanoparticle – Aqueous Solution Interface studied by Liquid-Microjet Photoemission

Hebatallah Ali[1], Bernd Winter[2] and Robert Seidel[3,4]*

[1] *Physics Department, Women Faculty for Art, Science and Education, Ain Shams University, Heliopolis, 11757 Cairo, Egypt*

[2] *Molecular Physics Department, Fritz-Haber-Institut der Max-Planck-Gesellschaft, Faradayweg 4-6, D-14195 Berlin, Germany*

[3] *Helmholtz-Zentrum Berlin für Materialien und Energie, Hahn-Meitner-Platz 1, 14109 Berlin, Germany.*

[4] *Department of Chemistry, Humboldt-Universität zu Berlin, Brook-Taylor-Straße 2, 12489 Berlin, Germany.*

**Corresponding author: Robert.Seidel@helmholtz-berlin.de*

1. Nitrogen-1s PEY-XAS and N 1s PES data for TiO_2 [NH_4^+] NPs

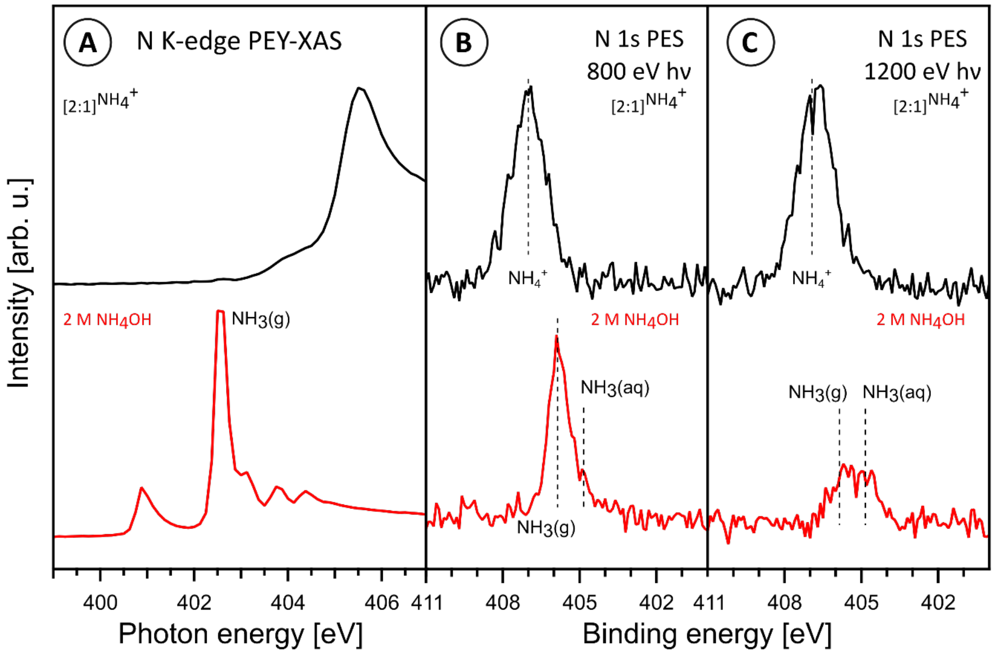

Figure S1: (A) Nitrogen K-edge partial electron yield X-ray absorption spectra of $[2{:}1]^{NH4+}$ TiO_2 NPs (black) and 2 M NH_4OH (red) aqueous solutions, and the corresponding N 1s photoelectron spectra measured at (B) 800 eV and (C) 1200 eV photon energies. Spectral contributions of NH_3(g), NH_3(aq), and NH_4^+ are labeled. Reproduced with permission from ref [1] (copyright 2020 American Chemical Society).

Figure S1A shows the nitrogen-1s PEY-XA spectra of the $[2:1]^{NH4+}$ sample, i.e. 50% of all titanium surface sites are covered by NH_4^+, and of a 2 M NH_4OH (pH~12) aqueous solution. The spectra were obtained by integrating the signal intensity of the valence band photoelectron spectra measured in 100 meV steps for photon energies between 391 eV and 407 eV. The absorption spectrum of the 2 M NH_4OH aqueous solution is dominated by NH_3 gas signal. This is due to the aforementioned chemical equilibrium between ammonium and ammonia, which leads to high concentrations of ammonia and a high volatility of the solution.[2] The N 1s PEY-XA spectrum of the $[2:1]^{NH4+}$ solution is similar to a reported spectrum of dry NH_4^+ salts[3]. We also measured (off-resonant) N 1s photoelectron spectra from the same solutions using 800 eV and 1200 eV photon energies (see Figures S1B and S1C). While the gas-phase peak at 405.9 eV binding energy dominates the spectrum at 800 eV photon energy, the same peak is of equal intensity with the liquid phase N 1s peak at 404.8 eV binding energy in the 1200-eV spectrum. The different intensities for the liquid NH_3 peak can be explained by the different probing depths, being larger for the 1200-eV photon energy. The $[2:1]^{NH4+}$ solution spectra for both photon energies exhibit a single N 1s peak from NH_4^+ at 407 eV binding energy (Figure S1B and S1C). We note that the change in the probing depth does not affect the peak shape and position.

2. Resonant photoelectron spectra of α-Fe_2O_3 NPs aqueous solutions

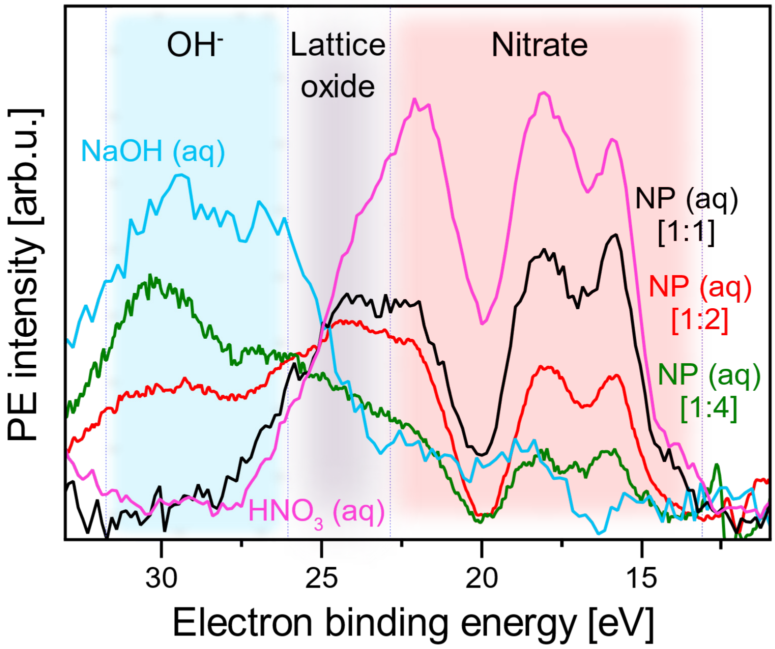

Figure S2: Oxygen-1s resonant photoelectron spectra measured at 532.2 eV from three a-Fe_2O_3 NPs aqueous solutions, as well as from 0.5 M HNO_3 and 0.5 M NaOH (measured at 532.8 eV) aqueous solutions. In all cases an off-resonant photoelectron spectrum measured at lower photon energies has been subtracted. Results for the following NP solutions are shown: purple: 0.5 M HNO_3. Black: 5 wt% NPs in 0.1 M HNO_3 [1:1]. Red: 10 wt% NPs in 0.1 M HNO_3 [1:2]. Green: 10 wt% NPs in 0.05 M HNO_3 [1:4]. The additional spectrum from 0.5 M NaOH is shown in blue. The shaded areas mark spectral regions which are dominated by contributions from a single species: blue region: OH^-. Grey region: lattice oxide. Red region: NO_3^-. In square brackets the ratios of number of adsorbed NO_3^- to number of available surface sites of the hematite NPs are shown. We observe spectral fingerprints of adsorbed hydroxide when not all iron surface sites are covered by NO_3^-. Reproduced with permission from ref [4] (copyright 2018 Royal Society of Chemistry).

Figure S2 shows the resonant valence band spectra, measured at a photon energy of 532.2 eV, of three NP solutions differing in the ratio of nitrate to free surface sites. An off-resonant spectrum measured at slightly lower photon energy was subtracted for each solution, highlighting the spectral features that are enhanced, which are resonant Auger peaks following O 1s → valence band excitations. One important observation is that the resonant spectrum from the [1:1] solution, i.e. all iron surface sites are covered by NO_3^-, is very similar to a resonance valence band spectrum from a 0.5 M HNO_3 aqueous solution (purple curve in figure S2), measured at 532.8 eV photon energy, where an off-resonant spectrum was subtracted. This means that the electronic structure of NO_3^-(aq) does not change upon adsorption on the hematite NP surface. The 4-peak structure can be attributed to characteristic spectator and participator resonant Auger peaks of NO_3^-. As the number of available H_2O adsorption sites on the hematite NP surface increases, the shape of the [1:2] solution spectrum (red curve in figure S2) and even more so the [1:4] solution spectrum (green curve in figure S2) changes and more closely resemble the resonance spectrum of a 0.5 M NaOH solution, measured at 532.8 eV with an off-resonant valence band spectrum subtracted (blue curve in figure S2). Comparing the blue spectrum with the green spectrum, we conclude that the resonance peaks in the [1:4] solution spectrum are spectral fingerprints of adsorbed hydroxide. We do not expect any significant energy differences between free and adsorbed hydroxide. It is argued that apparent differences in all spectral intensities result mainly from the different relative ratios of adsorbed nitrate to hydroxide.

3. PEY-XAS for Hematite NPs

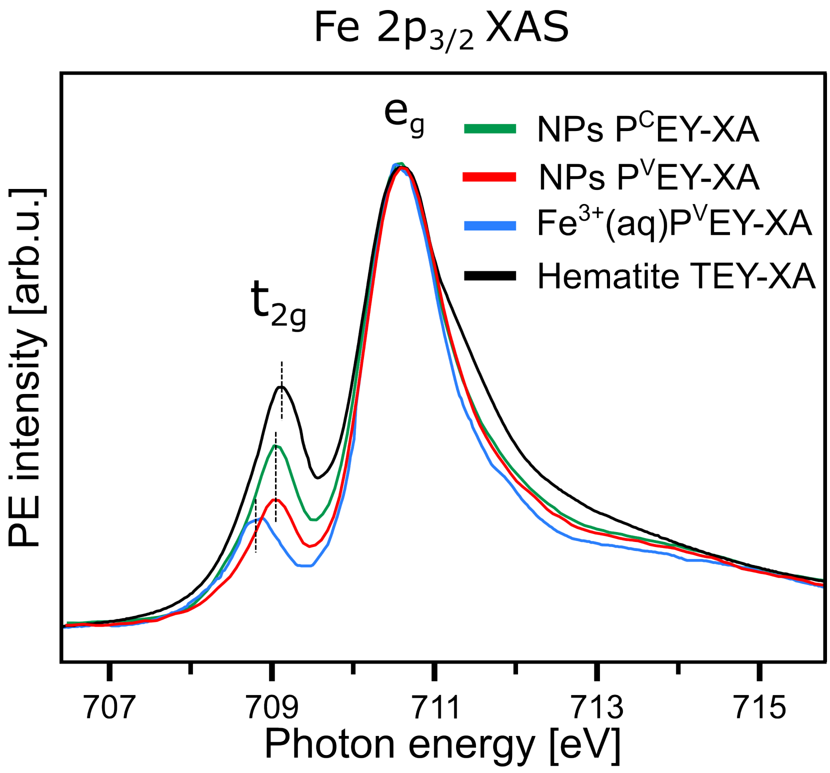

Figure S3: Iron L_3-edge P^CEY- and P^VEY-XA spectra (in green and red) of 10 wt% α-Fe_2O_3 NPs in 0.1 M HNO_3 aqueous solution, iron L_3-edge P^VEY-XA spectrum from 1 M $FeCl_3$ aqueous solution (in blue),[5] and a total-electron-yield spectrum from a solid hematite sample (in black).[6] Spectra were normalized to the absorption band at 710.5 eV photon energy. Reproduced with permission from ref [4] (copyright 2018 Royal Society of Chemistry).

The Fe $2p_{3/2}$ absorption spectra are also sensitive to the interfacial structure and (quantitatively) to the ligand field. The energy difference between the two peaks in Figure S3 is in first approximation similar to the difference in binding energy between the e_g and t_{2g} level, which is called 10 Dq. The 10 Dq values differ for the aqueous hematite NPs (1.5 eV), the monomeric Fe^{3+} salt solution (1.8 eV,[5]), and the solid hematite crystal (1.38 eV,[6]). We can exclude any nanoparticle size effects as an identical value of 1.38 eV has been reported in the literature for solid hematite and for dry hematite nanoparticles of 8 nm and 30 nm.[7] Since the 10Dq value is affected by the surrounding oxygen ligands, we conclude that the detected spectral signals originate from the NPs–aqueous interface and not from the NPs interior. Therefore, the detected Fe^{3+} ions in the NPs(aq) chemically interact with the aqueous oxygen species (NO_3^-, OH^-) and the hematite lattice oxide. Similarly, we can explain the intensity variation between 711 eV and 714 eV photon energy, which is dominated by spectral fingerprints of excited-state charge transfer.[8] The intensities in this region follow the same trend as the t_{2g} absorption intensity between 708 eV and 710 eV photon energy, although the effect is negligibly small when comparing P^VEY to P^CEY, which indicates that the ground-state iron t_{2g}–oxygen 2p orbital overlap is only little affected by the electron excited into higher lying states. The observed larger intensity near 712 eV absorption energy for crystalline hematite compared to the NP solutions and especially to Fe^{3+}(aq) suggests that ground-state charge transfer from the ligand to the iron cation is less in solution, implying a smaller orbital overlap. Water or hydroxide, either adsorbed on the nanoparticle surface or within the first hydration shell in the case of Fe^{3+}(aq) monomers, have a lower charge-transfer probability compared to O^{2-} ligands in bulk hematite.

4. References

1. Ali, H.; Golnak, R.; Seidel, R.; Winter, B.; Xiao, J., In-Situ X-Ray Spectroscopy of the Electric Double Layer around TiO_2 Nanoparticles Dispersed in Aqueous Solution: Implications for H_2 Generation. *ACS Applied Nano Materials* **2020**, *3*, 264-273.
2. Emerson, K.; Russo, R. C.; Lund, R. E.; Thurston, R. V., Aqueous Ammonia Equilibrium Calculations: Effect of PH and Temperature. *Journal of the Fisheries Board of Canada* **1975**, *32*, 2379-2383.
3. Ogunremi, T.; Cutler, J.; Christensen, C.; Sparks, C. In *X-Ray Absorption Spectroscopic Analysis of Raw and Processed Hog Manure*, Soils and Crops Workshop, 2007.
4. Ali, H.; Seidel, R.; Pohl, M. N.; Winter, B., Molecular Species Forming at the α-Fe_2O_3 Nanoparticle–Aqueous Solution Interface. *Chemical Science* **2018**, *9*, 4511-4523.
5. Thürmer, S.; Seidel, R.; Eberhardt, W.; Bradforth, S. E.; Winter, B., Ultrafast Hybridization Screening in Fe^{3+} Aqueous Solution. *Journal of the American Chemical Society* **2011**, *133*, 12528-12535.
6. Todd, E.; Sherman, D.; Purton, J., Surface Oxidation of Pyrite under Ambient Atmospheric and Aqueous (pH = 2 to 10) Conditions: Electronic Structure and Mineralogy from X-Ray Absorption Spectroscopy. *Geochimica et Cosmochimica Acta* **2003**, *67*, 881-893.
7. Gilbert, B.; Frandsen, C.; Maxey, E.; Sherman, D., Band-Gap Measurements of Bulk and Nanoscale Hematite by Soft X-Ray Spectroscopy. *Physical Review B* **2009**, *79*, 035108.
8. De Groot, F.; Kotani, A., *Core Level Spectroscopy of Solids*; CRC press, 2008.
